# The effect of sex and dietary crude protein level on nutrient transporter gene expression and cecal microbiota populations in broiler chickens

**DOI:** 10.1016/j.psj.2023.103268

**Published:** 2023-11-07

**Authors:** Ashley D. England, Sara de las Heras-Saldana, Kosar Gharib-Naseri, Sarbast K. Kheravii, Shu-Biao Wu

**Affiliations:** ⁎ School of Environmental and Rural Science, University of New England, Armidale NSW 2351, Australia; † Animal Genetics and Breeding Unit, School of Environmental and Rural Science, University of New England, Armidale NSW 2351, Australia

**Keywords:** gut microbiota, gene expression, sex effect, reduced crude protein, broiler chicken

## Abstract

It is well known that male and female broilers differ in their growth performance and that many physiological factors contribute to this difference. The aim of this experiment is to investigate if there are differences between male and female broilers in cecal microbiota and nutrient transporter gene expression and if these differences play a role in the growth performance of broilers. The possible effect of protein level and its interaction with sex on microbiota and expression of the nutrient transporters were also investigated. Samples were collected from male and female birds fed either standard crude protein (**SCP**) or reduced crude protein diets (**RCP**) at the age of d 35. The experiment was designed as a 2 × 2 factorial arrangement of treatments consisting of 448 Cobb 500 broilers assigned to 32-floor pens with 4 treatments, 8 replicates, and 14 birds per pen for performance measurements. The factors were sex (male or female) and dietary crude protein (**CP**) level (standard or reduced). Body weight gain (**BWG**), feed intake and feed conversion ratio were recorded for each pen. Sex had a significant effect on BWG and FCR (*P* < 0.001) where males had a significantly higher BWG and better FCR compared to females. There was a significant interaction between sex and protein level on feed intake (**FI**) (*P* < 0.05), where male birds had a higher FI compared to female birds only when the birds were fed SCP but not RCP diets. There was a significant interaction between CP level and sex on the expression of *CAT2* (*P* = 0.02) and *PEPT2* (*P* = 0.026) where the genes were significantly upregulated in females but only when the RCP diet was fed. The RCP diet upregulated the expression of *B^o^AT* (*P* = 0.03) as a main effect. Female birds had significantly higher expression of the *PepT-2* gene compared to the males. The alpha diversity of the cecal microbiota showed differences among the treatments. The Shannon diversity index was statistically higher (*P* = 0.036) for males fed the SCP diet and the Chao1 index for evenness was statistically higher (*P* = 0.027) in females fed the SCP diet. There was also a difference in the relative abundance of the 15 most common genera found in the cecal content of the broilers in this experiment and lastly, the differential composition of microbiota between the different treatments was also significantly different.

This study suggests that chickens are able to compensate for a reduction in AA substrates when fed a low CP diet through the upregulation of certain AA transporters, females may adapt to low CP diets better by such upregulation compared to males, and lastly, sex has an effect on the cecal microbial population and these differences contribute towards the performance differences between male and female broilers.

## INTRODUCTION

The small intestine of animals is where most of the feed digestion and nutrient absorption takes place. The digestion of different feed ingredients is related to a complex microbial ecosystem in the gut which is influenced by factors such as genotype (Turnbaugh et al., 2006), diet composition (Torok et al., 2013), feed additives (Singh et al., 2013), age (Niu et al., 2015) and health status of the animal (Wu et al., 2014). A few studies determined the effect of microbiota composition on production and health in chickens (Kohl, 2012; Stanley et al., 2012; Yeoman et al., 2012). It has been reported that genetic changes associated with improved weight gain and feed efficiency have resulted in changes to the gut physiology and gut microbial community composition of birds (Lumpkins et al., 2010). There appears to be a link between bird performance and gut microbiota composition, and with known performance differences between male and female chickens, we can assume that there would be a difference in the composition of gut microbiota between the sexes. A study conducted by Lumpkins et al. (2008) revealed that intestinal microbiota communities showed less than 30% similarity between male and female broilers. Torok et al. (2013) found that the gender of broilers significantly influenced the number of cecal Lactobacilli with males having higher numbers of *Ligilactobacillus salivarius* and *Lactobacillus crispatus.* Currently, there is a limited understanding of the functional capabilities of most microbial species found in the gastrointestinal tract of broilers as the intestinal microbiome of chickens is very complex and consists of varying bacterial populations (Apajalahti et al., 2004), and metabolomics research in chickens is still very limited to reveal the functionality of particular microbiota composition.

Digestion and absorptive capacities of the intestine in broilers are also known to affect performance (Ravindran and Abdollahi, 2021). It is thought that a potential difference in nutrient uptake in the intestine between male and female broilers could result in this difference in growth rate and thus a difference in final body weight between the 2 sexes. After digestion has taken place, the uptake of nutrients is controlled by transporter proteins which are located at the brush border of the intestinal epithelia. Brush border enzymes, Na+ -dependent neutral AA transporters, Na+ dependent neutral/cationic AA exchangers, and glucose transporters in the intestinal epithelium are closely associated with intestinal nutrient absorption capacity (Fotiadis et al., 2013; Hediger et al., 2013). Brush border enzymes include aminopeptidase N (**APN**) and sucrase-isomaltase (**SI**); Na+ dependent neutral AA transporters include B^o^AT and alanine, serine, cysteine and threonine transporter-1 (**ASCT1**); Na+ dependent neutral/cationic AA exchangers include Y+L AA transporter-1 (**y + LAT1**) and Y+L AA transporter-2 (**y +LAT2**) and the glucose transporters include GLUT-2 and GLUT-5. The majority of AA are transported by the PepT1 transporter either as free AA or peptides (Smith et al., 2013). Other AA transporters include EAAT3, rBAT, b^o^,+AT, CAT-1, CAT-2, and LAT-1, which are involved in the efflux of neutral, cationic, and branched chain and aromatic AA into the blood (Fotiadis et al., 2013; Kanai et al., 2013).

Few studies have been conducted to determine the differences in the expression of these transporters and digestive enzymes between male and female broiler chickens. Mott et al. (2008) reported that female chickens had an earlier peak in PepT1 gene expression as well as a greater expression of *SGLT1* and *EAAT3* genes compared to males. Weintraut et al. (2016) found a difference in the gene expression of AA and monosaccharide transporters between male and female turkeys with all genes, except *GLUT2* and *SGLT1*, being expressed greater in females. *GLUT2* was expressed at the same level in males as females and *SGLT1* was expressed greater in males. In comparison, Kaminski and Wong (2017) profiled the mRNA expression of an aminopeptidase and selected AA and monosaccharide transporters in the small intestine of male and female Aviagen line chickens on the day of hatch, d 7 and d 14. The expression of *b^o^,+AT, EAAT3, ASCT1, y+LAT2*, and *GLUT2* mRNA was greater in male than female chickens. The study found a sex × age interaction for *b^o^,+AT, PepT1, SGLT1, ASCT1*, and *y+LAT2* mRNA, with greater mRNA abundance in males than females at the day of hatch but no difference between males and females was detected at d 7 and d 14.

In this experiment 2 different levels of crude protein (**CP**) were used to detect variation in performance and thus potential differences in nutrient transporter gene expression and gut microbiota composition in male and female birds. Hernandez et al. (2012) reported that diets containing reduced CP poorly affected the performance of male broiler but not females. Most studies evaluating the effect of low CP diets on bird performance (Van Harn et al., 2019; Chrystal et al., 2020) indicated that moderate reductions in CP are enough to show variations in growth performance but further reduction of CP, over 30 g/kg, has been shown to compromise performance and this may negatively impact the health of the bird due to a potential deficiency in nonessential amino acids which are not supplemented in the diet (Dean et al., 2006; Kidd et al., 2021).

The objective of this study was to determine the differences in gut microbiota populations between male and female broilers with specific emphasis on the alpha diversity and evenness, relative abundance of the 15 most common genus, a description on shared and unique amplicon sequence variants (**ASVs**) and lastly the differential composition of microbiota between the different treatments. In addition, we investigated the effect of sex on nutrient transporter gene expression in broilers and the possible effect of protein level and its interaction with sex on microbiota and expression of the nutrient transporters.

## MATERIALS AND METHODS

### Ethics Statement

This experiment was approved by the Animal Ethics Committee of the University of New England (Approval No. AEC20-004). All broiler management procedures including health care, husbandry and use of laboratory animals fulfilled the requirements of the Australian Code for the Care and Use of Animals for Scientific Purposes (NHMRC, 2004).

### Experimental Design and Bird Management

A study was conducted to determine the effect of bird sex and CP level on growth performance, nutrient transporter gene expression and apparent amino acid digestibility in broilers. In this study, day-old mixed-sex Cobb-500 broiler chickens were obtained from Baiada hatchery in Tamworth, NSW, Australia. Upon arrival, all birds were weighed, vent sexed and allocated to floor pens according to treatments. This study was designed as a 2 × 2 factorial arrangement of treatments consisting of 448 birds assigned to 32-floor pens with 4 treatments replicated 8 times, each with 14 birds per pen. The factors were sex (male or female) and dietary CP level (standard or reduced). All the pens were located in the same environmentally controlled facility, equipped with feeders and nipple drinkers. The birds had ad libitum access to feed and water. The lighting, relative humidity and temperature were maintained following Cobb 500 guidelines (Cobb500 2018).

### Diets and Bird Performance

The ingredients and nutrient composition of the diets used in this experiment are shown in Table 1. The diets were formulated based on wheat and soybean meal. The standard crude protein (**SCP**) diets were formulated to meet the CP levels in the Cobb 500 guidelines (Cobb500 2018) (starter 22%, grower 20%, and finisher 19%) and the reduced crude protein (**RCP**) diets were formulated to have 2% less CP compared to the SCP diets (starter 20%, grower 18%, and finisher 17%). The nitrogen content of the diets was also analyzed using a combustion analyzer (Leco model FP-2000 N analyzer, Leco Corp., St. Joseph, Michigan, MI) and the analyzed CP content of the diets are shown in Table 1. The reduction in the RCP diet was obtained with additional L-valine, L-isoleucine and L-arginine, which were added to the diets to ensure the amino acid levels in both treatments were the same. Phytase was included in all treatments at 0.01% (Quantum Blue, AB vista Feed Ingredients, Marlborough, United Kingdom). Body weight gain (**BWG**), feed intake (**FI**) and mortality adjusted feed conversion ratio (**FCR**) were calculated for the finisher phase (d 25 to 35).

**Table 1:** Composition and nutritional content of the experimental diets.

	Starter (d 0 to 10)		Grower (d 11 to 24)		Finisher (d 25 to 35)	
Item	SCP	RCP	SCP	RCP	SCP	RCP
Ingredients (DM basis), %						
Wheat	53.2	58.0	42.6	52.2	41.6	48.8
Soybean meal	25.6	21.0	26.4	25.0	22.2	15.1
Sorghum	15.0	15.0	25.0	16.9	30.0	30.0
Canola oil	1.37	0.94	2.51	1.19	2.90	2.02
Limestone (fine)	1.18	1.19	1.08	1.13	1.02	1.04
Dical Phos 18P/21Ca	0.89	0.97	0.70	0.75	0.58	0.62
Sodium bicarbonate	0.32	0.45	0.02	0.41	0.15	0.44
Titanium dioxide	ˉ	ˉ	0.50	0.50	0.50	0.50
L-lysine HCl 78.4	0.54	0.68	0.27	0.54	0.29	0.46
DL-methionine	0.39	0.42	0.27	0.33	0.23	0.27
L-Arginine	0.53	0.33	ˉ	0.22	ˉ	0.16
L-threonine	0.35	0.27	0.08	0.20	0.13	0.16
L-Valine	0.16	0.24	ˉ	0.16	ˉ	0.10
L-Isoleucine	0.12	0.20	ˉ	0.14	ˉ	0.05
NaCl	0.110	0.040	0.273	0.003	0.138	0.007
Trace mineral premix^1^	0.110	0.110	0.110	0.110	0.110	0.110
Vitamin premix^2^	0.085	0.085	0.085	0.085	0.085	0.085
Choline Cl 70%	0.048	0.080	0.053	0.080	0.069	0.090
Phytase	0.010	0.010	0.010	0.010	0.010	0.010
Xylanase	0.005	0.005	0.005	0.005	0.005	0.005
Nutrients, %						
AMEn, kcal/kg	3,005	3,009	3,075	3,075	3,147	3,150
**Crude Protein (calculated)**	21.6	19.7	19.7	17.7	18.6	16.7
**Crude Protein (analysed)**	21.2	19.5	19.3	17.0	18.4	16.1
Crude fat	3.48	3.08	4.67	3.41	5.11	4.26
Crude Fiber	2.49	2.42	2.51	2.37	2.45	2.34
Dig. Arg	1.62	1.31	1.12	1.12	1.03	1.02
Dig. Lys	1.28	1.28	1.08	1.08	0.995	0.97
Dig. Met	0.65	0.66	0.54	0.55	0.48	0.49
Dig. Ile	0.87	0.87	0.77	0.75	0.70	0.64
Dig. Val	1.01	1.01	0.88	0.86	0.81	0.78
Calcium	0.90	0.91	0.82	0.83	0.76	0.76
Av. P	0.45	0.46	0.41	0.41	0.38	0.38
Choline, mg/kg	1,700	1,801	1,700	1,700	1,700	1,700
Linoleic 18:2	1.26	1.16	1.60	1.27	1.72	1.50

Abbreviations: CP = crude protein; SCP = standard protein; RCP = reduced protein.

1 Vitamin premix supplied the following per kilogram of diet: retinol, 12,000 IU; cholecalciferol, 5,000 IU; tocopheryl acetate, 75 mg, menadione, 3 mg; thiamine, 3 mg; riboflavin, 8 mg; niacin, 55 mg; pantothenate, 13 mg; pyridoxine, 5 mg; folate, 2 mg; scyanocobalamin, 16 mg; biotin, 200 mg; cereal-based carrier, 149 mg; mineral oil, 2.5 mg.

2 Trace mineral premix supplied the following per kilogram of diet: Cu (sulfate), 16 mg; Fe (sulfate), 40 mg; I (iodide), 1.25 mg; Se (selenate), 0.3 mg; Mn (sulfate and oxide), 120 mg; Zn (sulfate and oxide), 100 mg; cereal-based carrier, 128 mg; mineral oil, 3.75 mg.

### Sample Collection

On d 35, two birds from each pen were randomly selected and euthanised to collect jejunal tissue for gene expression assay and cecal contents for microbiota analysis. The birds were electrically stunned and then killed by cervical dislocation prior to being dissected. After dissecting the chickens, approximately 2 cm of proximal jejunal tissue was excised, flushed with PBS (4°C) and collected in 2 mL Eppendorf tubes filled with RNAlater solution (Ambion Inc, Austin, TX) and kept at 4°C for 8 h, and then stored at −20°C until required. The caecum was also excised and the cecal contents were collected from the sampled birds for bacterial quantification and stored at −20°C until required for analysis.

### Extraction of Cecal Bacterial DNA

The DNA of frozen cecal samples collected on d 35 was extracted using PowerFecal QIAcube HT Kit (Qiagen, Inc., Hilden, Germany) with modifications. Approximately 100 mg of cecal samples and 300 mg of glass beads (0.1 mm) were placed in a 2 mL Eppendorf tube. Later, 500 mL prewarmed PW1 was pipetted to Eppendorf tubes containing samples and placed into Tissuelyser II for 5 min at a frequency of 30 times per second to disrupt bacterial cells. The samples were incubated for 15 min at 90°C followed by centrifugation at 20,000 × *g* for 1 min. An aliquot of 400 mL supernatant was mixed with 150 ml of Buffer C3. The mixture was placed into the refrigerator at 4°C and incubated for 5 min before centrifuging at 20,000 × *g* for 1 min. The supernatant was transferred into a loading block (S-block) containing 20 mL Proteinase K and placed at room temperature for 10 min. Then the extraction was performed using the QIAcube HT followed by the manufacturer's instructions. The quantity and quality of the resulting DNA samples were determined on a Nanodrop 8000 spectrophotometer (Nanodrop Technologies, Wilmington, DE). DNA with standard ratios A260/A280 being >1.8 were recognized as of high purity and kept at −20°C until required.

### 16S rRNA Gene Sequencing and Bioinformatic Analysis

The V3-V4 regions of 16S rRNA genes were amplified with forward primer 341f and reverse primer 805r using polymerase. Sequencing was performed on an Illumina MiSeq system (2 × 300 bp) at Ramaciotti centre for genomics, UNSW, Sydney NSW 2052, Australia. The quality of the sequence reads was checked with fastQC v0.11.9 (Babraham Institute, Cambridge, United Kingdom) (Andrews, 2010). A quality control evaluation and cleaning of the reads were performed via the Quantitative Insights Into Microbial Ecology (QIIME V2021.8) software with the denoise (DADA2) function obtaining the Amplicon Sequence Variants (**ASV**). The Greengenes (13_8 99%) (De Santis et al., 2006) taxonomic classifier was used together with a pretrained naïve Bayes machine-learning classifier (Bokulich et al., 2018) to identify the taxa present in the samples. Alpha diversity indexes (Chao, Shannon, Simpson and Fisher) with a pairwise Wilcoxon test were evaluated in phyloseq v1.38 (McMurdie and Holmes, 2013). The effect of the group on the Faith's phylogenetic diversity and Pielou's evenness was assessed in QIIME2 with a Krustal–Wallis pairwise comparison. A differential microbial composition analysis was performed with DESeq2 v1.34.0 package (Love et al., 2014). Pair comparisons were performed between the groups (sex × diet) to identify the taxa that were significantly different (False discovery rate; FDR < 0.05). The differences in microbiota in birds with low or high weight gain (from the mean) were evaluated independently for the males and females including the diet in the model. The shared taxa between the groups of sex × diet were visualized with R using the venn v1.10 package.

### RNA Extraction and cDNA Synthesis

Total RNA from each jejunal sample was extracted using RNeasy Mini Kit (Qiagen, Inc.). The quantity and purity of the samples were measured with NanoDrop ND-8000 spectrophotometer (Thermo Fisher Scientific, Waltham, MA), and integrity with the Agilent 2100 Bioanalyzer and Agilent RNA 6000 Nano kit (Agilent Technologies Inc., Santa Clara, CA). The samples were accepted as high-quality if the value of 260/230 was > 1.8, 260/280 value was between 2.0 to 2.2, and the RIN number of each sample was higher than 7.0. The extracted RNA of each sample was reverse transcribed with the QuantiTect Reverse Transcription Kit (Qiagen, Inc.) according to the manufacturer's instructions. The Rotorgene Q real-time PCR machine (Qiagen, Inc.) was used to convert RNA into cDNA. The cDNA was diluted 10 times with nuclease-free water and stored at −20°C until required.

### Real-Time Quantitative PCR

The primers used in this experiment were either sourced from literature or newly designed as shown in Table 2. The specificity of primers was analyzed by PCR with a subpopulation of samples and fragments separated on Agilent 2100 Bioanalyzer using Agilent DNA 1000 Kit (Agilent Technologies Inc.). Quantitative PCR was performed in duplicates using an SYBR Green kit SensiFAST SYBR No-ROX (Bioline, Sydney, Australia) with Rotorgene Q real time PCR machine (Qiagen, Inc.). The PCR reaction was performed in a volume of 10 μL (microliter) containing 5 μL of 2 × SensiFAST, 400 mM of each primer and 2 μL of 10 × diluted cDNA template. The relative quantity of mRNA of the target genes was calculated by qBase+ version 3.0 (Biogazelle, Zwijnbeke, Belgium) software with *ACTB* and *GAPDH* as reference genes that were optimised from 10 widely used house-keeping genes prior to the analysis of target genes. The qBase+ applied an arithmetic mean method to transform logarithmic Cq value to linear relative quantity using the exponential function for relative quantification of target genes (Hellemans et al., 2007, Vandesompele et al., 2002). The output data were exported to SPSS Statistics package version 22 (IBM Corporation, Armonk, NY) for further analysis. The genes used for expression analysis in the jejunal tissue are as listed: *aminopeptidase N* (***APN***), *alanine, serine, cysteine and threonine transporter-1* (***ASCT1***), *b0,+amino acid transporter* (***b^o^,+AT***), *neutral amino acid transporter* (***B^o^AT***), *cationic amino acid transporter-1* (***CAT1***), *cationic amino acid transporter-2* (***CAT2***), *excitatory amino acid transporter-3* (***EAAT3***), *glucose transporter-2* (***GLUT2***), *glucose transporter-5* (***GLUT5***), *large neutral amino acid transporter-1* (***LAT1***), *peptide transporter-1* (***PepT1***), *peptide transporter-2* (***PepT2***), *sucraseisomaltase* (***SI***), *neutral and basic amino acid transport protein* (***rBAT***), *Y+L amino acid transporter-1* (***y + LAT1***) and *Y+L amino acid transporter-2* (***y + LAT2***).

**Table 2:** Sequences of primers used for quantitative real-time PCR.

Gene	Accession N^0^	Sequence	Size (pb)	Annealing T^0^	References
APN	NM_001013611.2	F-AATACGCGCTCGAGAAAACC R-AGCGGGTACGCCGTGTT	70	60	Gilbert et al. (2007)
ASCT1	XM 001232899.4	F-TTGGCCGGGAAGGAGAAG R-AGACCATAGTTGCCTCATTGAATG	63	60	Paris and Wong (2013)
B^o,+^AT	NM_001199133.1	F-CAGTAGTGAATTCTCTGAGTGTGAAGCT R-GCAATGATTGCCACAACTACCA	88	60	Gilbert et al. (2007)
B^o^AT	XM_419056.5	F-GTGTTTGGAACCCTAAATACGAGG R-TAGCATAGACCCAGCCAGGA	72	60	Kheravii et al. (2018)
CAT1	XM_015277945.1	F-CAAGAGGAAAACTCCAGTAATTGCA R- AAGTCGAAGAGGAAGGCCATAA	75	60	Gilbert et al. (2007)
CAT2	XM_015285435.1	F-TGCTCGCGTTCCCAAGA R- GGCCCACAGTTCACCAACAG	67	60	Gilbert et al. (2007)
EAAT3	XM_424930.5	F-TGCTGCTTTGGATTCCAGTGT R-AGCAATGACTGTAGTGCAGAAGTAATATATG	79	60	Su et al. (2014)
GLUT2	NM_207178.1	F-TGATCGTGGCACTGATGGTT R-CCACCAGGAAGACGGAGATA	171	60	Kheravii et al. (2018)
GLUT5	XM_417596	F-TCCAATAGCATGTCCGATGA R-GGAGGTTGAGGGCCAAAGTC	192	60	Gilbert et al. (2007)
LAT1	KT876067.1	F-GATTGCAACGGGTGATGTGA R- CCCCACACCCACTTTTGTTT	70	60	Gilbert et al. (2007)
PEPT1	AY029615.1	F-TACGCATACTGTCACCATCA R-TCCTGAGAACGGACTGTAAT	205	60	Guo et al. (2014)
PEPT2	NM_001319028.1	F-TGACTGGGCATCGGAACAA R-ACCCGTGTCACCATTTTAACCT	63	60	Paris and Wong (2013)
SI	XM_015291762.1	F-GCTTTAAGATGGGCAAGAGGAAG R- CCACCACCAGGCAAAAGAGG	65	60	Kheravii et al. (2018)
rBAT	XM_426125.4	F-CCCGCCGTTCAACAAGAG R- AATTAAATCCATCGACTCCTTTGC	70	60	Gilbert et al. (2007)
Y + LAT1	XM_418326.5	F-TACTGAGGCTGACTGGAGGAA R- ACGACGTACAGCACAATATCTGG	227	62	Kheravii et al. (2018)
Y + LAT2	NM_001005832.1	F-GCCCTGTCAGTAAATCAGACAAGA R-TTCAGTTGCATTGTGTTTTGGTT	82	60	Gilbert et al. (2007)

### Statistical Analysis

Data were tested for normality using the Shapiro–Wilk test in SPSS which was > 0.05 for both gene expression and performance data. The normally distributed data were analyzed according to a 2 × 2 factorial arrangement of treatments, using the General Linear Model procedure of SPSS Statistics package version 22 (IBM Corporation) to assess the main effects of sex and CP level, and their interactions. Mean values of the treatments were compared within the confidence interval adjusted by Tukey's range test when the interaction was significant. Significant differences were determined at *P* < 0.05, and *P* values between 0.05 and 0.10 were reported as a tendency of significance. The microbiota data was not normally distributed and therefore the diversity and evenness were analyzed using the nonparametric Kruskal–Wallis test.

## RESULTS AND DISCUSSION

### Broiler Performance

The effects of sex and dietary CP level on broiler performance in the period from d 25 to 35 are presented in Table 3. Sex had a significant effect on BWG and FCR (*P* < 0.001) where males had a significantly higher BWG and better FCR compared to females. No interactions between sex and CP level were observed for BWG and FCR. There was a significant interaction between sex and protein level on FI (*P* < 0.05), where male birds had a higher FI compared to female birds only when the birds were fed SCP but not RCP diets.

**Table 3:** Performance of broiler chickens from d 25 to 35 in response to different rearing methods and dietary protein level.

Sex	Protein level	BWG	FI	FCR
Male	SCP	1,215	2,125^a^	1.749
	RCP	1,155	2,025^ab^	1.756
Female	SCP	1,028	1,901^b^^s^	1.848
	RCP	1,041	2,026^ab^	1.946
SEM		21.8	45.45	0.027
Main effects				
Sex				
Female		1,035^b^	1,963^b^	1.897^a^
Male		1,185^a^	2,075^a^	1.753^b^
SEM		15.45	32.15	0.019
Diet				
RCP		1,098	2,006	1.851
SCP		1,122	2,021	1.799
SEM		15.45	32.15	0.019
*P*-value				
Sex		< 0.001	0.020	< 0.001
Protein level		0.294	0.787	0.059
Sex × protein level		0.105	0.019	0.106

Abbreviations: CP = crude protein; SCP = standard protein; RCP = reduced protein.

BWG = body weight gain per bird; FI = feed intake per bird; FCR= feed conversion ratio.

a,b Means in a column not sharing the same superscripts are significantly different according to the Tukey test (*P* < 0.05).

### Gene Expression of Nutrient Transporters and Digestive Enzymes

The effect of dietary CP level and sex on gene expression of nutrient transporters and digestive enzymes are presented in Tables 4 and 5. There was a significant interaction between CP level and sex on the expression of *CAT2* (*P* = 0.02) and *PEPT2* (*P* = 0.026) where the genes were significantly upregulated in females only when the RCP diet was fed. On the other hand, RCP diet only upregulated these 2 genes in female but not in male birds. There was a tendency toward a significant interaction between CP level and sex on the expression of *ASCTI1* (*P* = 0.073), where again, the gene was upregulated in females but only when the RCP diet was fed. It has been shown by Hernandez et al. (2012) that a 1.5% reduction in dietary CP fed to Ross 308 broilers adversely affected the performance of the male birds, from d 22 to 35, but not the female birds, whose CP could be reduced to 3% with no adverse effects. Similar diet, wheat-soybean-based, was used in this study. Hence, the reason for this could be that females are better able to compensate for the reduction in nonessential AA by upregulating the expression of certain AA transporters to a greater extent compared to males.

**Table 4:** The effect of dietary CP level and sex on the gene expression of B^o^AT, CAT2, PepT2, y+LAT2, ASCTI1, SI, and y+LAT1 in jejunal tissue of broilers on d 35.

Sex	Protein level	B^o^AT	CAT2	PepT2	y+LAT2	ASCT1	SI	y+LAT1
Male	SCP	0.854	1.131^a^	1.578^a^	1.017	1.040^a^	0.762	0.923
	RCP	1.434	0.848^a^	0.953^a^	1.189	0.978^a^	1.305	1.198
Female	SCP	0.710	0.637^a^	1.326^a^	0.763	0.703^a^	1.017	0.985
	RCP	1.202	1.998^b^	4.522^b^	1.236	1.790^b^	1.168	1.262
SEM		0.232	0.330	0.799	0.165	0.304	0.190	0.155
Main effects								
Gender								
Female		0.956	1.317	2.924	1.000	1.247	1.093	1.124
Male		1.144	0.990	1.265	1.103	1.009	1.033	1.061
SEM		0.164	0.234	0.565	0.117	0.216	0.134	0.109
Diet								
RCP		1.318	1.423	2.737	1.213	1.384	1.236	1.230
SCP		0.782	0.884	1.452	0.890	0.871	0.889	0.954
SEM		0.164	0.234	0.565	0.117	0.216	0.134	0.109
*P*-value								
Sex		0.426	0.331	0.049	0.539	0.443	0.757	0.688
Protein level		0.030	0.116	0.122	0.063	0.107	0.081	0.087
Sex × protein level		0.852	0.020	0.026	0.374	0.073	0.314	0.996

**Table 5:** The effect of dietary CP level and sex on the gene expression of APN, EAAT3, GLUT2, LAT1, PepT1, b^o^+AT, rBAT, CAT1, and GLUT5 in jejunal tissue of broilers on d 35.

Sex	Protein level	APN	EAAT3	GLUT2	LAT1	PepT1	b^o^+AT	rBAT	CAT1	GLUT5
Male	SCP	1.363	1.179	0.914	1.098	0.933	0.800	0.775	1.160	0.896
	RCP	1.094	1.180	1.505	1.458	1.262	1.214	1.184	1.096	1.153
Female	SCP	0.940	1.001	1.120	0.811	0.987	1.142	1.037	1.178	1.328
	RCP	1.183	1.209	1.188	1.046	0.968	1.038	1.093	1.097	1.217
SEM		0.313	0.244	0.304	0.231	0.208	0.238	0.153	0.207	0.232
Main effects										
Gender										
Female		1.062	1.105	1.154	0.928	0.977	1.090	1.065	1.138	1.272
Male		1.229	1.180	1.209	1.278	1.098	1.007	0.980	1.128	1.024
SEM		0.221	0.173	0.215	0.164	0.147	0.169	0.108	0.147	0.164
Diet										
RCP		1.139	1.194	1.35	1.25	1.115	1.126	1.138	1.096	1.185
SCP		1.151	1.090	1.02	0.96	0.960	0.971	0.906	1.169	1.112
SEM		0.221	0.173	0.215	0.164	0.147	0.169	0.108	0.147	0.164
*P*-value										
Sex		0.600	0.762	0.858	0.144	0.569	0.730	0.580	0.962	0.294
Protein level		0.970	0.673	0.289	0.212	0.465	0.522	0.140	0.728	0.755
Sex × protein level		0.420	0.674	0.398	0.789	0.412	0.288	0.257	0.967	0.434

Crude protein level had a significant effect on the expression of *B^O^AT* (*P* = 0.03) and a tendency towards having a significant effect on the expression of *y+LAT1* (*P* = 0.087), *y+LAT2* (*P* = 0.063), and *SI* (*P* = 0.081). All these genes were upregulated in the birds fed the RCP diet. In fact, the expression of all the nutrient transporters and the enzymes was upregulated numerically in birds fed the RCP diet except for *APN* and *CAT-1*. This may not be by chance but has implications on biological functions. The B^0^AT transports a broad range of neutral amino acids which account for 15 of standard amino acids as nutrients. The upregulation of this gene may have a profound effect in the utilization of the amino acids. In addition, y+LAT1 (SLC7A7) and y+LAT2 (SLC7A6) both function as obligatory exchangers of cationic amino acids and neutral amino acids. Their tendency to be higher in the RCP group may also aid to the AA utilization. These results give suggestive evidence that feeding a diet low in CP results in the upregulation of AA transporters which may indicate a physiological adaptation in order to compensate for the reduction in AA substrates. Corzo et al. (2011) also found that the expression of certain AA transporters were increased when Ross 308 broilers were fed RCP diets which supports our findings.

Sex as a main effect only affected (*P* = 0.049) the expression of the *PepT-2* gene which was upregulated in females compared to males. This is not in agreement with the results reported by Mott et al. (2008) who observed that White Plymouth Rock female chickens had an earlier peak in *PepT1* gene expression as well as a greater expression of *EAAT3* genes compared to males. Weintraut et al. (2016) found a difference in the gene expression of AA and monosaccharide transporters between male and female turkeys with all genes, except *GLUT2* and *SGLT1*, being expressed greater in females, where *GLUT2* was expressed at the same level in males as females and *SGLT1* was expressed greater in males. In comparison, Kaminski and Wong (2017) profiled the mRNA expression of an aminopeptidase and selected AA and monosaccharide transporters in the small intestine of male and female Aviagen line chickens fed corn-soybean-based diet and found the expression of *B^o^,+AT, EAAT3, ASCT1, y+LAT2*, and *GLUT2* mRNA was greater in male than female chickens. We speculate that these conflicting results may be due to genetics, growth stages, age, breed, nutrients, and locations where the genes are expressed all of which may play important roles in the effect of sex on nutrient transporter gene expression. As shown in the studies, different breeds and diet types were used, and the age of the birds that were sampled from as well as the examined tissue for the gene expression also differed. Therefore, future studies are warranted to better understand the effect of sex on nutrient transporter gene expression and its interactions with other factors such as nutrition, age, genetics and the locations where the genes are expressed.

### Cecal Microbiota

**Figure 1:**
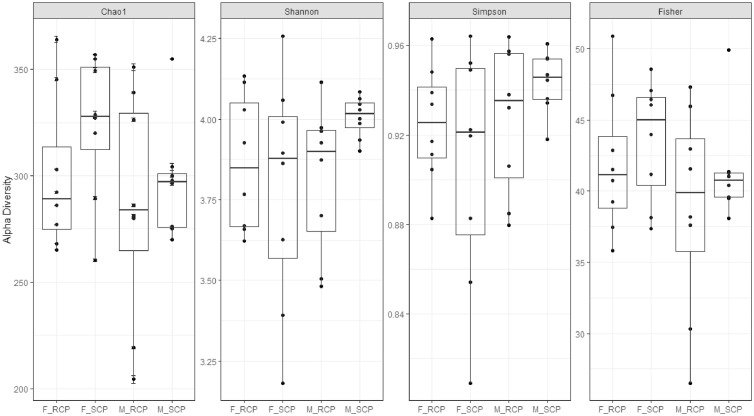
Diversity of microbiota in the cecal content on d 35 showing alpha diversity by treatment (sex × diet) F_RCP = Females fed reduced protein diet; F_SCP = Females fed standard protein diet; M_RCP = Males fed reduced protein diet; M_SCP = Males fed standard protein diet.

Figure 1 shows the alpha diversity and evenness of the microbiota in the cecal content on d 35. Alpha diversity analysis at the genus level showed that the Shannon diversity index was statistically significant amongst the treatments. The microbiota diversity in the caecum of males fed the SCP diet was significantly higher (*P* = 0.036) compared to the other treatments. The Chao1 index for evenness was statistically higher (*P* = 0.027) in females fed the SCP diet (Table 6).

**Table 6:** Krustal-Wallis pairwise comparison testing the Faith's phylogenetic diversity and Pielou's evenness of microbiota in the cecal content of broilers on d 35.

Group 1	Group 2	H	*P*-value	q-value
F_RCP (n = 8)	F_SCP (n = 8)	4.864	0.027	0.107
	M_RCP (n = 8)	1.335	0.248	0.440
	M_SCP (n = 8)	0.044	0.834	0.834
F_SCP (n = 8)	M_RCP (n = 8)	0.276	0.600	0.719
	M_SCP (n = 8)	4.412	0.036	0.107
M_RCP (n = 8)	M_SCP (n = 8)	1.103	0.294	0.440

F_RCP = Females fed reduced protein diet; F_SCP = Females fed standard protein diet.

M_RCP = Males fed reduced protein diet; M_SCP = Males fed standard protein diet.

**Figure 2:**
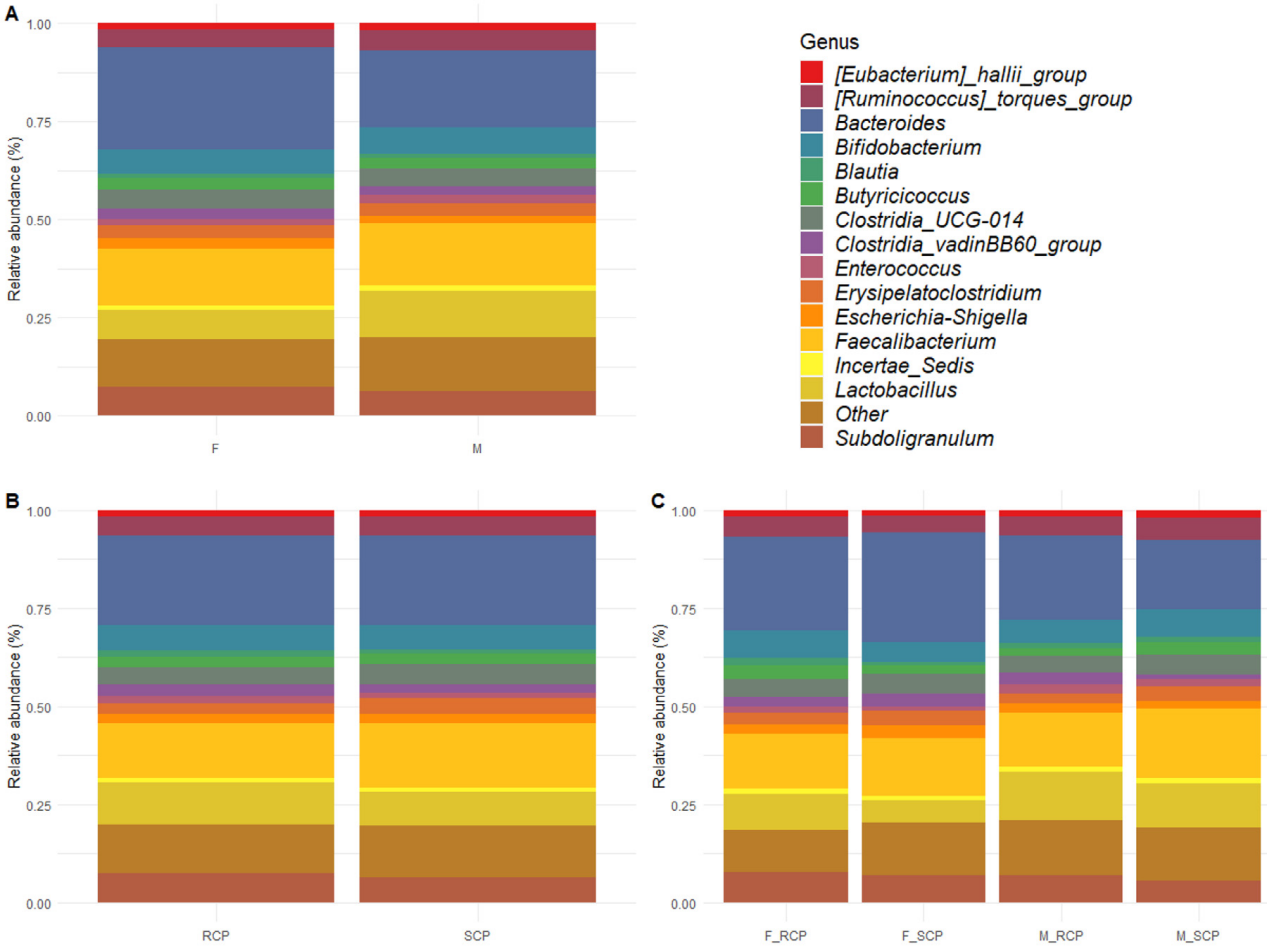
Relative cecal microbiota abundance of the 15 most abundant genus on d 35 by (A) Sex, (B) Diet, and (C) sex × diet. F = Female; M = Male; RCP = Reduced crude protein; SCP = Standard crude protein; F_RCP = Females fed reduced protein diet; F_SCP = Females fed standard protein diet; M_RCP = Males fed reduced protein diet; M_SCP = Males fed standard protein diet.

The effect of sex and CP level on the relative abundance of the 15 most abundant genera found in the cecal content of the broilers in this study is shown in Figure 2. At the genus level, *Bacteriodes* (Bacteriodetes Phylum) was the most abundant, followed by the genus *Faecalibacterium* (Firmicutes Phylum) and then the genus *Lactobacillus* (Firmicutes Phylum). This is in agreement with other studies that found Bacteriodetes and Firmicutes among the most predominant microbes found in the ceca of chickens (Torok et al., 2011; Wei et al., 2013; Choi et al., 2014; Lee et al., 2017). These bacteria are known to have the ability to degrade indigestible fiber in the GIT (Lee et al., 2017).

**Figure 3:**
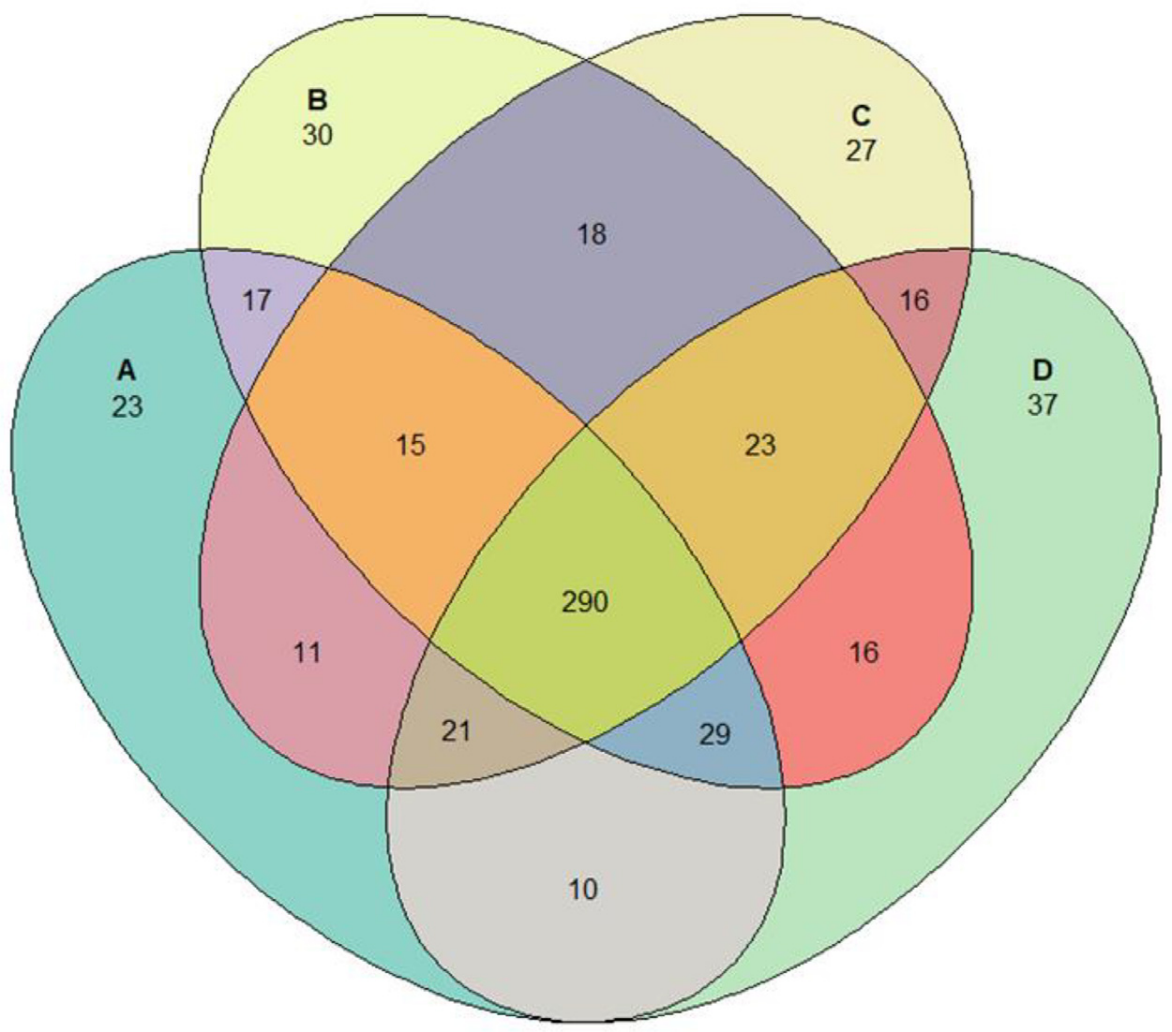
Venn diagram illustrating shared and unique ASVs for filtered taxa among (A) M_SCP, (B) F_RCP, (C) M_RCP, (D) F_SCP. F_RCP = Females fed reduced protein diet; F_SCP = Females fed standard protein diet; M_RCP = Males fed reduced protein diet; M_SCP = Males fed standard protein diet

Further differences were observed in the abundances of ASVs between treatments. Figure 3 illustrates the total number of ASVs shared by the treatments as well as the number of ASVs that are unique to each treatment. The Venn diagram identified 290 ASVs that were shared between all treatments. The cecal content of males fed the SCP had the lowest number of unique ASVs with 23 whilst the cecal content of females fed the SCP diet had the highest number of unique ASVs with 37. A total of 500 ASVs were found in the cecal content of male birds, of which 337 were shared between birds fed the SCP and RCP diets. A total of 522 ASVs were found in the cecal content of female birds, of which 358 were shared between birds fed the SCP and RCP diets. It has been well documented that the gut microbiota profile can be significantly impacted by different factors, such as diet, drug, dietary supplement or feed additives, genetic background, age, sex and the interaction between different factors (De Cesare et al., 2019; Le Roy et al., 2019; Liu et al., 2020; Ricke, et al., 2020; Cui et al., 2021).

**Figure 4:**
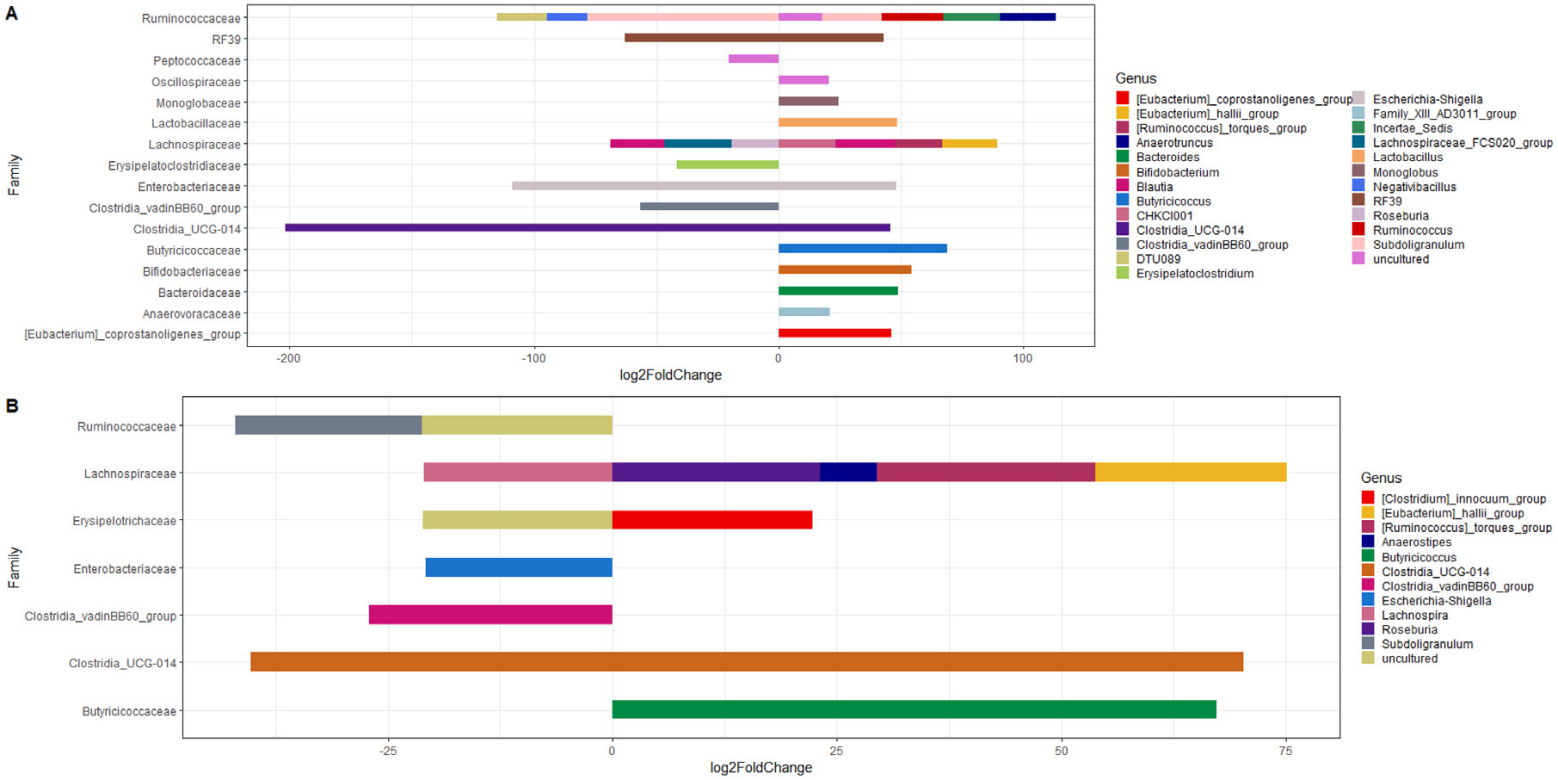
Differential microbiota composition for (A) male and (B) female birds with high and low weight gain.

**Figure 5:**
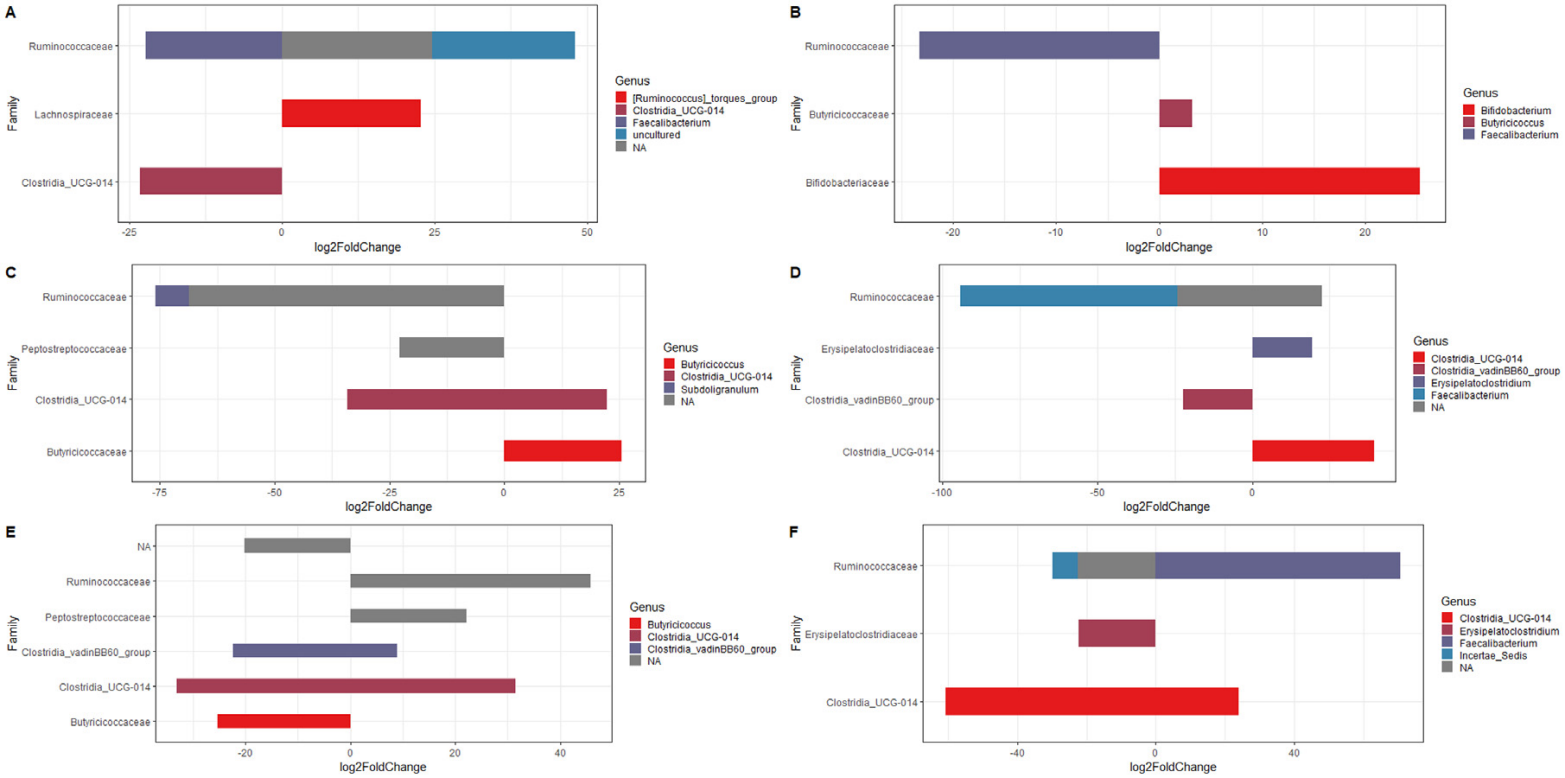
Differential composition of microbiota in the cecal content on d 35 for (A) Sex (F vs M), (B) Diet (SCP vs RCP), (C) M_SCP vs F_SCP, and (D) M_RCP vs F_RCP, (E) M_RCP vs M_SCP, (F) F_RCP vs F_SCP. F_RCP = Females fed reduced protein diet; F_SCP = Females fed standard protein diet; M_RCP = Males fed reduced protein diet; M_SCP = Males fed standard protein diet.

Generally, male chickens usually perform better than female chickens under same conditions, i.e., genetic background and diet, and there is evidence of essential role of gut microbiota in chicken's growth performance (Cui et al., 2021). The cecal content of females had a higher relative abundance of *Bacteriodes* compared to males whereas the cecal content of males had a higher relative abundance of *Lactobacillus* compared to females (Figure 2A). Lactobacillaceae members are known to play a positive role in improving gut health, immune characteristics and production performance (Joat et al., 2021) and the higher relative abundance found in males could contribute to their superior growth performance. Analyses on abundance differences in microbiota associated with performance were carried out, identifying 25 and 74 ASVs for female and male birds that were associated with high and low weight gain. Lactobacillaceae was significantly more abundant in higher weight gain male birds compare to birds with weights below the average weight gain (Figure 4A) while it was not significant in female birds (Figure 4B). Further differences were observed based on the results of the differential composition of the microbiota, the cecal content of female birds had a significantly lower composition of *Faecalibacterium* (FDR = 1.35E-11) and *Clostridia UCG-014* (FDR = 1.14E-12) and a significantly higher composition of *Ruminococcus torques* (FDR = 2.47E-12) compared to the cecal content of males (Figure 5A). *Clostridia UCG-014* has been found to activate the metabolic pathway associated with tryptophan and relieve gut inflammation (Yang et al., 2021) and a higher prevalence of *Faecalibacterium* was associated with higher BW (Lee et al., 2017) as this genus plays a major role in butyrate production (Rychlik, 2020). The bacteria *Ruminococcus torques* belongs to a group that is known to degrade gastrointestinal mucin (Wilson et al., 1997), hence, weakening the protection provided by the intestinal mucous layer. Further, Cui et al. (2021) predicted the functions of bacteria in cecal contents of genders and PICRUSt analysis showed that the cecal microbiota in male chickens contributed in many metabolism processes, including protein digestion and absorption, glycan degradation, metabolism of D-Arginine, D-ornithine, vitamin B6, nicotinate and nicotinamide, glycosaminoglycan degradation, and transporters, while the cecal microbiota in female chickens was mainly participated in glycerophospholipid metabolism and transportation. Hence, the differences in cecal microbiota found between males and females might have contributed in some form to the differences in growth performance. However, further studies on both microbial profile and metagenomics at different ages are warranted to confirm if microbiota across the intestine play the vital roles in chicken performance with considering different factors such as age, sex, breed and diet composition.

The cecal content of birds fed the SCP diet had a slightly higher relative abundance of *Faecalibacterium* and *Erysipelatoclostridium* and a lower relative abundance of *Lactobacillus* compared to the cecal content of birds fed the RCP diet (Figure 2B). De Cesare et al. (2019) also found that the relative abundance of certain species changed in cecal contents of chicken (Ross 308) when diets in low CP were fed; more specifically, it was reported that birds fed standard protein diet had a slightly higher relative abundance of Firmicutes, including *Faecalibacterium*, at both d14 and 42. However, relative abundance of Lactobacillaceae significantly decreased in the low protein group at d14 in the ceca of broilers fed low protein diet but the same family significantly increased in the low protein group and at d 42. This indicates that the microbial profile can be changed by different factors such as age and sex of the birds. Further, statistically significant differences (FDR = 2.13E-12) were only found in the composition of Ruminococcaceae family for *Faecalibacterium* genus, which was lower in cecal content of birds fed SCP than birds fed RCP while higher composition of *Bifidobacterium* (FDR = 8.10E-15) and *Butyricicoccus* (FDR = 0.04) was observed in the cecal content of birds fed SCP compared to the birds fed RCP diet (Figure 5B). The changing in the composition of the 3 aforementioned genus by the diets might have not affected the performance of the birds significantly as all of them are considered commensal bacteria in the chicken gut and the diets might have created a favorable environment for these commensal bacteria to grow.

The cecal content of female birds fed the SCP diet had the lowest relative abundance of *Lactobacillus*, followed by females fed the RCP diet whilst the cecal content of males fed the RCP diet had the highest relative abundance of *Lactobacillus* (Figure 2C). The cecal content of females fed the SCP diet had the highest relative abundance of *Bacteriodes*, followed by females fed the RCP diet, and lastly, the cecal content of males fed the SCP diet had the lowest relative abundance of *Bacteriodes*. In contrast, Lee et al. (2017) found that male chickens had a higher relative abundance of *Bacteriodes*, but it has also been reported that the phylum *Bacteriodetes* is strongly associated with diet types and, therefore, their abundance can change depending on the feed ingredients used. The cecal content of males fed the SCP diet had the highest relative abundance of *Faecalibacterium*, followed by females fed the SCP diet, and lastly, the cecal content of males fed the RCP diet had the lowest relative abundance of *Faecalibacterium*. This genus is producer of short-chain fatty acids such as butyric acid and formic acid (Bjerrum et al., 2006) which may have an important function on growth performance (Garcia et al., 2007). In accordance with previous studies, our results suggest that the group of broilers that performed the best would have a higher relative abundance of *Faecalibacterium*, however, further studies confirming its association with performance are needed*.* The cecal content of both females fed the RCP diet and males fed the SCP diet had the highest relative abundance of *Bifidobacterium* followed by males fed the RCP diet and lastly, the cecal content of females fed the SCP diet had the lowest relative abundance of *Bifidobacterium. Bifidobacterium* is known for their ability to degrade simple carbohydrates and oligosaccharides (Apajalahti and Vienola, 2016) and the higher relative abundance in females fed the RCP diet may explain why these birds did not perform worse compared to females fed the SCP diet. Males fed the SCP diet also had a higher relative abundance of *Bifidobacterium* which could explain the better performance of these birds over others of the same sex but fed the diets associated with a lower relative abundance of *Bifidobacterium.*

A significantly higher composition of *Clostridia UCG-014* (FDR < 0.05) was observed in the cecal content of males fed the RCP compared to the cecal content of females fed the RCP diet (Figure 5D). In addition, the cecal content of males fed the RCP diet had a significantly lower composition of *Butyricicoccus* (FDR = 4.15E-07) compared to the cecal content of males fed the SCP diet (Figure 5E). The cecal content of females fed the RCP diet had a significantly higher composition of *Faecalibacterium* (FDR < 0.05) compared to the cecal content of females fed the SCP diet (Figure 5F). The potential performance-related bacteria identified in this study belong to the phylum *Firmicutes* which are involved in the production of short chain fatty acids (acetate, propionate, and butyrate) through the fermentation of dietary fiber. These short-chain fatty acids play a role in the regulation of intestinal health (Liu et al., 2021) and can therefore enhance broiler performance.

## CONCLUSIONS

The current study revealed that the majority of the nutrient transporter genes were upregulated in birds fed the RCP diet suggesting that the upregulation of AA transporters in birds fed a low CP diet may indicate a physiological adaptation in order to compensate for the reduction in AA substrates. Key differences in the relative abundance and composition of cecal microbiota between male and female broilers were also observed, specifically a difference in the relative abundance of *Bacteriodes, Lactobacillus, Faecalibacterium*, and *Bifidobacterium*. These bacteria are known to have the ability to degrade indigestible fiber in the GIT thus might have contributed to the performance differences between male and female broilers most likely through their different abilities to digest feed components. As this study was based on a single collection point, more research will be needed in the future to confirm whether the difference in enzyme and nutrient transporter gene expressions play a role in broiler performance and to understand the dynamics in microbial community structure and function.

## DISCLOSURES

We declare that we have no financial and personal relationships with other people or organizations that can inappropriately influence our work, there is no professional or other personal interest of any nature or kind in any product, service and/or company that could be construed as influencing the content of this paper.
